# Effectiveness of a recovery workshop implemented in community mental health services in Catalonia (Spain): study protocol for a non-randomized controlled trial

**DOI:** 10.1186/s12888-022-04350-y

**Published:** 2022-12-27

**Authors:** Hernán María Sampietro, Maite Barrios, Georgina Guilera, J. Emilio Rojo, Juana Gómez-Benito

**Affiliations:** 1 ActivaMent Catalunya Associació, Barcelona, Spain; 2 Department of Social Psychology and Quantitative Psychology, University of Barcelona, Passeig de la Vall d’Hebron, 171, 08035 Barcelona, Spain; 3 Group on Measurement Invariance and Analysis of Change (GEIMAC), Institute of Neurosciences, University of Barcelona, Barcelona, Spain; 4 Department of Medicine, International University of Catalonia, Barcelona, Spain; 5 Hospital Benito Menni CASM, Sisters Hospitallers, Sant Boi de Llobregat, Spain

**Keywords:** Mental Health, Recovery, Empowerment, Self-determination, Hope, Perceived Social Support

## Abstract

**Background:**

Many countries today are undergoing a paradigm shift in mental health policies towards a recovery-oriented and rights-based approach. From this perspective, self-determination and self-management are fundamental factors for recovery. Despite this shift, there is still a lack of evidence on the effectiveness of training programmes aimed at promoting self-determination and self-management in recovery processes implemented in southern European or Spanish-speaking countries. The aim of this paper is to present a study protocol that evaluates the effectiveness of a 12-session recovery workshop implemented in community mental health services in Catalonia (Spain).

**Methods/design:**

This is a 12-week follow-up multi-centre non-randomized controlled trial design. At least 160 users will be recruited from 13 Community Rehabilitation Services (CRS) in Catalonia. Eligible participants are adult (≥ 18 years old) users of a CRS, who sign a written consent to participate. The experimental group participates in a recovery workshop, in which people learn to develop and implement their own plan of personal recovery, which includes a Wellness Toolbox, a Maintenance Toolkit, a Personal Growth Plan, a Mirror of Relapses, a Crisis Plan, and a Learning Agenda. The control group participates in the usual activities of the CRS. Data is collected using a questionnaire of sociodemographic characteristics, personal recovery, empowerment, hope and perceived social support. The users’ measurements are taken at the baseline and one week after the end of the workshop. The primary outcome measures include the Self-Identified Stage of Recovery and the Maryland Assessment of Recovery in Serious Mental Illness Scale (short version). The secondary outcome measures include the Netherlands Empowerment List, Dispositional Hope Scale, and Multidimensional Scale of Perceived Social Support. Descriptive statistics for characterizing the sample size will be performed. Multivariate analyses for repeated measures designs will be used to evaluate the primary and secondary outcomes. Between-group and within-subject comparisons will be conducted.

**Discussion:**

The results of the study will provide information on the usefulness of recovery workshops in a Mediterranean cultural context. Additionally, if this workshop is effective, it will be proposed for inclusion within the portfolio of community mental health services in Catalonia.

**Trial Registration:**

ISRCTN11695542 (Registration date: 5 July 2022).

**Supplementary Information:**

The online version contains supplementary material available at 10.1186/s12888-022-04350-y.

## Background

Currently, public mental health policies in many countries around the world promote recovery-oriented care, an approach whose main objective is to help people to have a meaningful and satisfying life, in accordance with their own preferences and values, beyond the presence or absence of symptoms [1–3]. Meeting this goal presupposes that people are allowed to make their own decisions. In this respect, self-determination [4–6] and exercising citizenship rights [7] have been both identified as conditions for the possibility of personal recovery. According to this idea, mental health service providers have a responsibility to promote self-determination, providing people with the tools they need to direct their own recovery processes, making their own decisions [8].

In 2002, Connecticut (USA) was the first state mental health authority in the United States to adopt a policy aimed at promoting a recovery-oriented system of care [9]. Within a few years, a trend towards pro-recovery policies spread throughout the whole United States, and to Scotland, first, and then to the rest of the United Kingdom [10]. This mental health policy shift was replicated in most anglophone countries [11] and in northern Europe [12] over the course of the first decade of this century and, more recently, in some countries insouthern Europe, such as Italy [13] and Spain [14]. Gradually, more and more countries around the world are adopting a recovery-oriented approach, driven by the QualityRights initiative of the World Health Organization [15]. WHO QualityRights is a strategic line and a toolkit designed to “improve service delivery in line with a person-centred, recovery and human rights-based approach” (WHO, p.80) [16].

In Spain, the administration of the health system is overseen by the Autonomous Communities (Spanish regions with their own government). In this regard, Catalonia, one of the 17 regions of Spain, introduced the recovery-oriented and rights-based approach to care in its strategic plans in 2017 [17, 18], promoting the deployment of a whole series of initiatives aimed at generating a change in the model of mental health care. One such initiative is the *Activa’t per la Salut Mental* programme (Get Active for Mental Health) [19], developed jointly by the Mental Health Services Administration, relatives’ and users’ organizations, and survivors’ movements. The aim of the programme is to encourage and enable people with mental health problems and their families to become active health agents in their recovery processes.

Within the framework of this programme, a new recovery tool has been designed and developed based on the principles and objectives of recovery-oriented approaches. This tool has two components: the Manual for the Recovery and Self-Management of Well-Being [20] and the Personal Workbook [21], developed through a participatory process (in which around 300 people took part) using a mixed methodology. A workshop has also been designed to enable participants to use the tool effectively [22]. It is now necessary to evaluate the effectiveness of this training program and the materials in order to be able to promote their use in mental health services and associative movements.

A little over a decade ago, Silverstein and Bellack (2008) summarized twenty years of research on recovery, identifying four areas of research and development: (a) the definition of recovery; (b) the development of reliable measures of recovery; (c) rates of and barriers to recovery; and (d) the effectiveness of recovery-oriented care [23]. Nine years after that publication, Leonhart et al. (2017) found that, even though the volume of work on personal recovery had significantly increased, there are still big gaps needing further research [24]. One of these gaps is how the intervention programmes aimed at promoting personal recovery are being implemented and evaluated.

Filling this gap is particularly necessary in non-anglophone countries, in which very little research has been conducted on this topic. A recent scoping review found that, out of 37 published studies aimed at evaluating the effectiveness of a recovery workshop, only 7 had been conducted in non-English speaking countries, and all of these were in Asia (three in Palestine and the others in China, Korea, Japan and Pakistan) [25]. More significantly, one of these studies found no improvements for most of the variables evaluated after the intervention [26]. Likewise, only three studies focused on exploring the applicability of one of these tools, the Wellness Recovery Action Plan (WRAP) [27], for an ethnic minority [28–30], with two of them concluding that it is necessary to adapt this recovery tool to incorporate a critical analysis of oppression and a gender perspective in the workshops [29, 30].

In short, the review of previous literature highlights the need to analyse the applicability and evaluate the efficacy of this type of intervention in other cultural contexts because, to date, “most of the studies were conducted in high-income countries, with a recovery-oriented mental health system, in which there are well-established networks of users and survivors, and an Anglo-Saxon/Protestant cultural background that highlights individual freedom and self-determination” (Sampietro et al., p. 9) [25].

In this article, we present the study protocol of a non-randomized controlled trial designed to evaluate the effectiveness of the recovery workshop of the *Activa’t per la Salut Mental* programme, implemented in mental health community services in Catalonia, Spain.

The main objective of the study is to evaluate if the recovery workshop of the *Activa’t per la Salut Mental* programme has an impact in terms of an improvement in personal recovery. The secondary objective is to evaluate the changes in the level of empowerment, hope and perceived social support among participants of the recovery workshop of the *Activa’t per la Salut Mental* programme.

The primary hypothesis to be tested is that participants in the experimental groups (i.e., users of a 12-week recovery workshop implemented in mental health community rehabilitation services) will improve their personal recovery more than the participants in the control group (i.e., users of the standard activities of the mental health community rehabilitation services) immediately one week after the intervention.

The secondary hypothesis to be tested is that the participants in the experimental groups (i.e., users of a 12-week recovery workshop implemented in community rehabilitation services) will improve their empowerment, hope, and perceived social support more than the participants in the control group (i.e., users of the standard activities of the community rehabilitation services) immediately one week after the intervention.

## Method

This protocol has been drafted following the Standard Protocol Items: Recommendations for Interventional Trials (SPIRIT) Statement, for reporting a clinical trial protocol [31] (see Additional file 1 for checklist). This is the first published version of the protocol.

### Study design

This is a multi-centre non-randomized controlled trial comparing users of community mental health services who participate in a 12-week recovery workshop to users who do not.

This study is part of a biggest project entitled “Toward recovery in people diagnosed with a severe mental disorder: Definition, assessment and intervention (RECO-DAI)”.

### Study setting and CRS enrolment

The study is conducted in 13 mental health Community Rehabilitation Services (CRS), spread over 75% of the territory of Catalonia. Both large cities (such as Barcelona, with more than 1.6 million inhabitants) and small municipalities (such as Amposta, with just 20,000 inhabitants) have been included. A CRS is a free, public mental health care resource with the aim of comprehensively accompanying people throughout their own recovery process. They offer specialized care designed to promote functional recovery, strengthen psychosocial skills and facilitate people’s inclusion in the community.

The CRS enrolment was conducted eight months before users’ recruitment. Mental health service providers in Catalonia that had previously collaborated with the *Activa’t per la Salut Mental* programme were invited to participate. Together, these 13 CRS offer community mental health services to a region with a total population of 1,700,000 people.

Prior to the study, from 16 April to 5 October 2021, at least two professionals from each CRS received 20.5 h of training on: (a) what personal recovery is and how to promote it; (b) how to draft their own personal recovery plan (with the new materials created in Catalonia) [20, 21]; (c) how to implement the recovery workshop of the *Activa’t per la Salut Mental* programme [22]; and (d) how to evaluate this recovery workshop (including instruments and study design).

### Eligibility criteria and recruitment

Eligible participants are adults who take part in the activities of a CRS, and who consent to participate in the study. There are 15 potentially eligible CRS whose professionals had previously attended the recovery workshop training course for trainers. Of these, only the CRS that have agreed to participate in the research, allow accessibility, and commit to the continuity of the project have been or will be involved in this study.

The inclusion criteria for participants are: (a) aged over 18 years and under 65 years; (b) user of a CRS; and (c) signature of the informed consent and commitment to participate. The exclusion criteria are: (a) presence of relevant cognitive impairment and comprehension difficulties; and (b) presence of severe or decompensated somatic disease.

The CRS professionals oversee the recruitment, offering all users of the service meeting the inclusion criteria the option of participating in an evaluated recovery workshop as part of the range of activities offered by the service. All users interested in participating can sign up, up to the maximum capacity of 10 participants. If the maximum capacity of workshop participants is exceeded, the last people signed up will remain on a waiting list for future cycles of the workshop. This is the standard procedure for all regular CRS activities. For the control group, the CRS professionals will follow the same procedure. If there are more than 10 people interested in participating in the control group, the CRS professionals will carry out intentional sampling, taking into account the demographic variables (age, sex, level of education, etc.) of the experimental group, to form a relatively equivalent group with respect to these variables.

All users interested in participating in the study are invited to a session prior to the start of the workshop, in which the research team resolves any possible doubts about the study’s aim and procedures. Their written informed consent is also obtained in this session.

CRS professionals assign an alphanumeric code to all trial participants, both in the experimental group and in the control group. The members of the research team do not have access to the information related to the group to which the participants belong until the interpretation phase of the collected and processed data.

The recruitment period ran from January to October 2022.

### Sample size

A power analysis was conducted to determine the appropriate sample size. To do so, we used the G*Power software (Version 3.1.9.7) [32]. To detect differences in each group with repeated measures within-between factors (ANOVA) that correspond to a small effect size (0.18), with an alpha risk of 0.05, and a power of 80%, the adequate sample size required for this study in each group will be 64 participants. Assuming an attrition rate of 25% over the course of the three and a half months of the study, we estimate that a total sample of 160 users of a CRS will be required.

### Intervention

The recovery workshop of the *Activa’t per la Salut Mental* programme presents and trains how to use a series of materials that facilitate self-determination in developing one’s own recovery plan. These materials include:

- Manual for Recovery and Self-Management of Well-Being: This introduces the concept of personal recovery and provides information, guidance and a variety of strategies that individuals can use to develop their own recovery plan [20]. It organizes these resources and strategies based on whether they can be found in ourselves, in our immediate environment (relatives, friends, etc.), in the community, in the professional care network, or in mutual support environments. It offers links to web portals that help them find these resources in the region where they live (e.g., it allows access to the entire network of mutual aid groups in Catalonia).
- Personal workbook: This is a practical document that facilitates the organization and management of the strategies and resources that people have at their disposal to develop their own personalized recovery plan [21]. This material organizes the resources according to what they are used for: promoting well-being, having a life project, trying to avoid relapses, identifying the beginnings of a relapse, making advanced decisions to deal with mental health crises more easily, and learning from one’s own experience. In addition, the workbook offers sections to be filled in order to write down their own recovery plan.

The workshop is organized into 12 sessions, each 90 min long, which include conceptual content, participatory dynamics, and periods for collective reflection. Additionally, from the fourth session onwards, an initial quarter of an hour is dedicated to accompanying and/or sharing in groups the advances made by the workshop participants in the development of their personal recovery plan.

Among other specific contents, the participants of the Recovery Workshop learn: (a) the meaning of recovery from the recovery model perspective (i.e., their distinguishing characteristics, origin and principles) and the role of self-determination and self-management in recovery processes in mental health; (b) the importance of having a personalized recovery plan centred on the person and by the person, according to their own preferences and values, identifying their current needs and their own recovery goals, and the need to identify the resources people have within their reach to carry out their recovery process, including professional care resources, but also those they can find in themselves, in their immediate environment, in the community, and in mutual support spaces; (c) the value of well-being for mental health and the importance of including in the daily routine or in daily actions some activities that generate well-being (using the Wellness Toolbox section); (d) the necessity of agency in preventing mental health crises and the importance of identifying what should and should not be done in their own everyday lives to try to reduce the chances of having a relapse (using the Maintenance Toolkit section); (e) how to build or identify one’s own motivations and goals in life, and the importance of having a life project, something to live for (using the Personal Growth Plan section); (f) how to identify the first signs of a relapse (with or without the help of others) and the resources that each person may have available to respond to them (using the Mirror of Relapses section); (g) the importance of identifying who the support people are that one could count on in case of need, whether they are people from the family environment, friends, professionals or mutual support environments (using the Mirror of Relapses and Crisis Plan sections); (h) the need to prepare for possible future relapses or mental health crises, making an own advanced decisions plan (using the Crisis Plan section); (i) how to record and systematize one’s own experience to use it as a source of basic learning to guide their own recovery process (using the Learning Agenda section).

Moreover, a distinctive feature of the Workshop is that not only is it a space for learning and reflection in relation to these contents, but also that it has a practical orientation. People who complete the recovery workshop are expected to have developed their own Recovery and Self-Management of Well-Being Plan, progressing through and completing their Personal Workbook in collaboration with their environment and professional care resources.

The learning objectives and specific content of each session can be found in the Guide for Implementing the Workshops with the Manual for Recovery and Self-Management of Well-Being [22].

The recovery workshop is implemented by CRS professionals, who have previously participated in 20.5 h of training on: (a) the principles of recovery-oriented care; (b) the materials from the *Activa’t per la Salut Mental* recovery workshop; (c) the contents and activities of the Recovery Workshop; and (d) the design of the evaluation of the effectiveness of the recovery workshop study. Additionally, in sessions fifth and tenth of the workshop, experts with experience in the recovery process (users and survivors) participate as trainers. In the fifth session, they share a life story of recovery and, in the tenth session, they explain what participating in a space of mutual support has given them in terms of their recovery process. They also present the network of mutual support that the participants have within their reach.

For their part, the members of the control group participate in the usual CRS activities. These activities include development of cognitive, communication and emotional skills workshops; leisure and free times activities; strengthening of social relationships; training and guidance for job placement; etc. For the most part, these activities take place on a weekly basis or more frequently.

### Outcomes and participant timeline

The primary outcome of the study is personal recovery. The recovery stage will be measured in accordance with Retta Andresen’s proposal [33, 34], and the level of recovery will be measured in line with the proposal of Drapalski and Medoff [35, 36]. Two psychometric instruments are used to measure personal recovery:

#### Self-Identified Stage of Recovery (SISR)

This scale is based on the stages model of recovery, which identifies five stages in the recovery process: moratorium, awareness, preparation, rebuilding and growth [33]. This instrument is a two-part scale. The first (SISR-A) allows the stage of recovery to be assessed, and the second (SISR-B) measures four component processes of recovery (SISR-B). The SISR-A is a single-item, forced-choice measure, with five answer options (from A to E), each presenting one of the stages of the recovery process. The participants have to choose one of these items, identifying at which stage they consider that they currently are. The SISR-B is a 4-item scale, assessing four key component processes of recovery: finding hope, re-establishment of identity, finding meaning and taking responsibility. It uses a Likert scale with six response options, ranging from 1 (Disagree strongly), to 6 (Agree strongly). The total score is obtained from the sum of all the answers, ranging from 4 to 24. A higher score on the scale is indicative of a higher level of recovery.

#### Maryland Assessment of Recovery in Serious Mental Illness Scale – Short (MARS12)

The scale is designed to assess six of the central components of personal recovery, according to the Substance Abuse and Mental Health Services Administration (SAMHSA) definition [37], excluding the components that do not focus on the person but on the service system or community, and combining empowerment and self-direction. These six domains are: self-direction/empowerment, holistic, non-linear, strengths-based, responsibility, and hope. The original instrument is a 25-item scale [35, 36]. For this study, we use the short version, a 12-item scale (MARS12) developed by Deborah Medoff (2015) [38]. MARS12 uses an ordinal Likert scale with five response options, ranging from 1 (Not at all), to 5 (Very much). The total score is obtained from the sum of all the answers, ranging from 12 to 60. A higher score on the scale is indicative of a higher level of recovery.

The secondary outcomes of the study are empowerment, hope and perceived social support. Three psychometric instruments are used to measure these variables:

#### Netherlands Empowerment List (NEL)

This is designed to assess both personal and social dimensions of empowerment. The NEL is a 40-items scale that includes six subscales: (a) Confidence and purpose (12 items); (b) Social support (7 items); (c) Connectedness (6 items); (d) Self-management (5 items); (e) Caring community (6 items); and (f) Professional help (4 items) [39]. This instrument uses a Likert scale with five response options, ranging from 1 (Strongly disagree), to 5 (Strongly agree). The total score is obtained from the sum of all the answers, ranging from 40 to 200. A higher score on the scale is indicative of a higher level of empowerment.

#### Dispositional Hope Scale (DHS)

This is based on the two-factor model of hope, which includes pathways and agency [40]. The DHS is a 12-item scale, of which four are pathways items, four measure agency, and the remaining four are filler items. This instrument uses a Likert scale with four response options, ranging from 1 (Definitely false), to 4 (Definitely true). The total score is obtained from the sum of all the answers, ranging from 12 to 48. A higher score on the scale is indicative of a higher level of hope.

#### Multidimensional Scale of Perceived Social Support (MSPSS)

This scale is designed to assess perceived social support from three sources: family, friends, and a significant others [41]. MSPSS is a 12-item scale, with four items measuring each of the three sources of social support. This instrument uses a Likert scale with seven response options, ranging from 1 (Very strongly disagree) to 7 (Very strongly agree). The total score is obtained from the sum of all the answers, ranging from 12 to 84. A higher score on the scale is indicative of a higher level of perceived social support.

#### Sociodemographic characteristics

Sociodemographic characteristics are collected at the baseline of the study, and they include: age, gender, marital status, education level, working status, diagnostics, and coexistence unit.

(See Additional file 2 for the test battery)

Pre-intervention data collection is conducted around one week before the beginning of the recovery workshop at each CRS. Post-intervention data collection is conducted around one week after the end of the recovery workshop at each CRS.

**Fig. 1:**
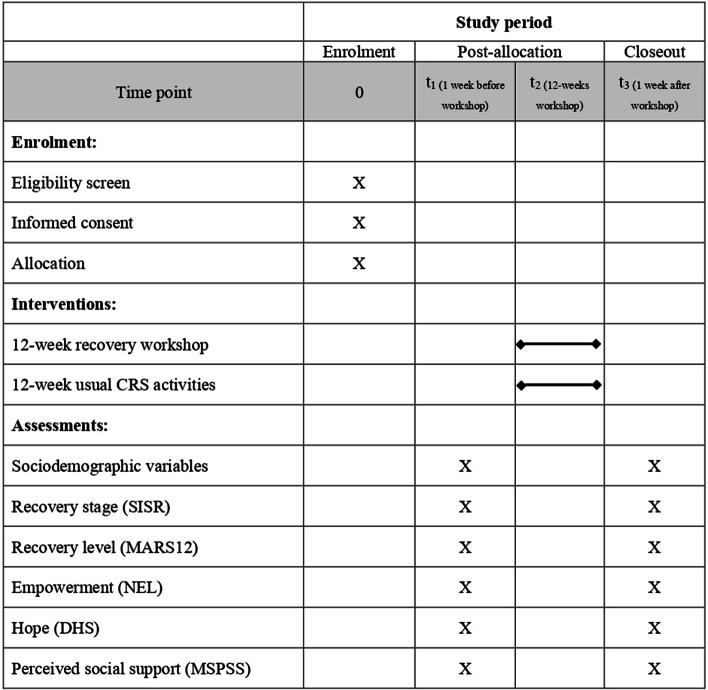
Schedule of enrolment, interventions, and assessment

SPIRIT Fig. 1 shows a summary of all the steps described above.

### Data storage and security

The members of the research team will be responsible for the treatment and custody of the data. The data will be collected using the Qualtrics digital platform, which has a data handling security certificate (DIN EN ISO/IEC 27001:2017). When the data collection is completed, the data will be downloaded to an institutional computer of the University of Barcelona belonging to one of the researchers and then immediately removed from the Qualtrics platform. The data files, to which access will be limited exclusively to the members of the team, will be stored and safeguarded on the institutional cloud (OneDrive UB).

The research team commit to comply with Regulation 2016/679 of the European Union, of 27 April, on the protection of natural persons with regard to the processing of personal data and on the free movement of such data, and the Spanish Organic Law 3/2018, of 5 December, on the protection of personal data and guarantee of digital rights.

### Statistical analysis

To evaluate the effectiveness of the *Activa’t per la Salut Mental* recovery workshop, descriptive statistics for characterizing the sample size will be performed. Multivariate analyses for repeated measure designs will be used for measures of personal recovery, empowerment, hope and perceived social support. Between-group and within-subject comparisons will be performed. In addition, ANOVA and ANCOVA will be used when comparing groups.

Statistical analyses will be performed using JASP software (Version 0.16.3) [42].

## Discussion

The aim of this paper is to describe a N-RCT regarding a recovery workshop being implemented in community mental health services in Catalonia, Spain.

The WRAP [27], a similar tool created in the USA, has been widely implemented and evaluated, and is currently recognized as an evidence-based intervention by the Substance Abuse and Mental Health Service Administration [43]. In contrast, there is little evidence regarding the efficacy of this type of interventions in non-English-speaking contexts and no study has been found in southern European or Spanish-speaking countries [25].

This study will provide data on the effectiveness of the recovery workshop of the *Activa’t per la Salut Mental* programme. Moreover, this effectiveness will be measured with psychometric instruments that assess variables of the CHIME model [11], with a perspective of recovery-oriented care. This differentiates it from the majority of previous studies, whose most evaluated variables are symptom improvement and knowledge, and attitudes towards recovery [25]. Furthermore, the psychometric instruments used in this study to assess personal recovery [33, 35] and empowerment [38] have been created by or with the participation of users and survivors as part of the research team. In this respect, these instruments share the philosophy of the paradigm shift. This is a perspective that recognizes users of mental health services as subjects of knowledge (i.e., experts by experience) and not as objects of an intervention.

In addition, this study will be the first to provide data of the effectiveness of a group recovery workshop designed to promote self-determination in the recovery process, in a Mediterranean cultural context, with a Latin language, a Catholic tradition and a characteristic family and cultural environment.

Finally, a workshop intended to promote personal recovery is implemented for the first time in the community rehabilitation services in Catalonia. This evaluation is conducted in order to assess the possible inclusion of the workshop into the portfolio of mental health services in our country, as has happened with other previous projects of the *Activa’t per la Salut Mental* programme.

The study protocol has two limitations that should be pointed out. Firstly, we have chosen a non-randomized trial design to do the study. Therefore, outcome evaluation could not be double-blinded. The main reason is that we wanted to include small cities in the sample as well. These populations have a smaller CRS, with fewer places offered and few users. Taking into account the fact that participation in the activities of the CRS is voluntary, there might be not enough people interested in participating in a 12-week training workshop to create a randomized control group. A pre-recruitment consultation revealed that this was the case for at least 3 CRS. Therefore, there is an evident bias, as the people in the experimental group are those interested in participating in a workshop aimed at promoting personal recovery, and the people in the control group are those not interested in this activity.

Secondly, the loss of subjects over the course of the 12-week implementation of the recovery workshop may cause a possible decrease in the statistical power and a selection bias. As this is a training workshop, offered as a voluntary activity, being implemented for the first time at the CRS, of which people had no prior references with respect to what it would be like to participate, it is expected that there will be a higher rate of abandonment of the activity than the average for the usual activities of the CRS. We expect an abandonment rate of around 20-25%. The possible bias risk here is that the people who complete the workshop and the evaluation are those who are most interested in or identify more with what the training offers them.

## Supplementary Information

**Additional file 1.** SPIRIT Checklist.

**Additional file 2.** Test battery.

## Data Availability

The dataset generated and analysed in this study will be available from the corresponding author. Results generated in this study will be published in peer-reviewed journals and presented at national/international conferences. The materials used in the recovery workshop are publicly accessible at https://activatperlasalutmental.org/lactivat/el-circuit-activat/psicoeducacio/#recuperacio: (1) Manual for Recovery and Self-Management of Well-Being; (2) Personal Workbook; (3) Guide to Implementing the Workshops.

## Abbreviations

N-RCT: Non-Randomized Controlled Trial
CRS: Community Rehabilitation Service
SISR: Self-Identified Stage of Recovery
MARS12: Maryland Assessment of Recovery in Serious Mental Illness Scale – Short
NEL: Netherlands Empowerment List
DHS: Dispositional Hope Scale
MSPSS: Multidimensional Scale of Perceived Social Support

## Declarations

All participants of the study sign an informed consent before participating.
