# Supplementary material for: Effectiveness of a recovery workshop implemented in community mental health services in Catalonia (Spain): study protocol for a non-randomized controlled trial
Source: BMC Psychiatry. 2022 Dec 27;22:827. doi: 10.1186/s12888-022-04350-y (PMC9793523; doi:10.1186/s12888-022-04350-y)
Supplement: Supplementary file 2 — Additional file 2. Test battery. [file 12888_2022_4350_MOESM2_ESM.docx]

**Código de Identificación del/la Participante:** __________________________

(tres últimas cifras y letra del DNI)

Por favor, a continuación, completa la información o bien señala con una "x" la opción que mejor se corresponda a ti.

**Fecha de hoy** (día/mes/año)**:** _____/_____/_____ **Población:** ______________________

**Nombre del SRC:** _____________________________________________________________

**Edad:** ________

| **Género:**   \| 1. \| Hombre \|  \| \| --- \| --- \| --- \| \| 2. \| Mujer \|  \| \| 3. \| No binario \|  \| \| 4. \| No contesta \|  \| | **¿Tienes un diagnóstico de trastorno mental?**   \| Sí \|  \|  \| No \|  \|  \| No sé \|  \| \| --- \| --- \| --- \| --- \| --- \| --- \| --- \| --- \|   **¿Cuál/cuáles?**  _______________________________________  _______________________________________  _______________________________________ |
| --- | --- | --- | --- | --- | --- | --- | --- | --- | --- | --- | --- | --- | --- | --- | --- | --- | --- | --- | --- | --- | --- |

| **Estado civil actual:**   \| 1. \| Casado/a o en pareja \|  \| \| --- \| --- \| --- \| \| 2. \| Soltero/a \|  \| \| 3. \| Separado/a Divorciado/a \|  \| \| 4. \| Viudo/a \|  \| | **Nivel de estudios finalizado:**   \| 1. \| Sin estudios \|  \| \| --- \| --- \| --- \| \| 2. \| Primarios \|  \| \| 3. \| Secundarios \|  \| \| 4. \| Universitarios \|  \| |
| --- | --- | --- | --- | --- | --- | --- | --- | --- | --- | --- | --- | --- | --- | --- | --- | --- | --- | --- | --- | --- | --- | --- | --- | --- | --- |

| **¿Con quién vives?**   \| 1. \| Familia de origen (madre/padre) \|  \| \| --- \| --- \| --- \| \| 2. \| Familia propia (pareja/hijos) \|  \| \| 3. \| Solo/Sola \|  \| \| 4. \| Comparto piso \|  \| \| 5. \| Centro Institucional \|  \| \| 6. \| Otro (especificar): \|  \| \|  \|  \|  \| | **Situación laboral** (puedes señalar más de una):   \| 1. \| Estoy trabajando \|  \| \| --- \| --- \| --- \| \| 2. \| Estoy en el paro \|  \| \| 3. \| Estoy incapacitado (cobro pensión) \|  \| \| 4. \| Estoy buscando trabajo (sin prestación) \|  \| \| 5. \| Estoy estudiando \|  \| \| 6. \| Estoy jubilado/a \|  \| \| 7. \| Cuido de mi hogar y/o mi familia \|  \| \| 8. \| Otro (especificar): \|  \| \|  \|  \|  \| |
| --- | --- | --- | --- | --- | --- | --- | --- | --- | --- | --- | --- | --- | --- | --- | --- | --- | --- | --- | --- | --- | --- | --- | --- | --- | --- | --- | --- | --- | --- | --- | --- | --- | --- | --- | --- | --- | --- | --- | --- | --- | --- | --- | --- | --- | --- | --- | --- | --- | --- |

**Etapa de Recuperación Autopercibida (SISR).**

Las personas diagnosticadas de un problema de salud grave pueden variar su manera de sentir lo que es vivir con esa condición en diferentes momentos. A continuación, se muestran cinco afirmaciones que describen cómo las personas a veces pueden sentirse cuando viven con un problema de salud mental.

Por favor, **lee las cinco afirmaciones** (A-E) **antes de responder** a las siguientes preguntas.

| **A)** “No creo que las personas puedan recuperarse de un problema de salud mental. Siento que he perdido el control de mi vida y que no hay nada que pueda hacer para ayudarme a mí mismo/a". |  |
| --- | --- |
| **B)** “*Recientemente* me he dado cuenta de que las personas pueden recuperarse de un problema de salud mental grave. Estoy comenzando a pensar que quizá sea posible ayudarme a mí mismo/a.” |  |
| **C)** “Estoy *empezando* a aprender cómo puedo superar mi problema de salud mental. He decidido que voy a seguir adelante con mi vida.” |  |
| **D)** “*Actualmente* puedo manejar mis problemas de salud mental razonablemente bien. Lo estoy haciendo bien y me siento bastante optimista respecto al futuro.” |  |
| **E)** “Siento que *actualmente* tengo el control de mi salud y de mi vida. Lo estoy haciendo muy bien y el futuro parece prometedor.” |  |

De las cinco afirmaciones anteriores, ¿cuál dirías que describe ***mejor*** cómo te has sentido en el **último mes** en relación a vivir con tu problema de salud mental? Marca la casilla correspondiente a esa afirmación.

A continuación, se presentan cuatro afirmaciones sobre cómo pueden sentirse las personas en relación a ciertos aspectos de su vida.

Durante el **último mes**, ¿cuál es tu grado de acuerdo con cada afirmación? Marca el número correspondiente.

1. Estoy seguro de que encontraré formas de lograr mis metas en la vida.

| Totalmente en Desacuerdo | En Desacuerdo | Un poco en Desacuerdo | Un poco de Acuerdo | De Acuerdo | Totalmente de Acuerdo |
| --- | --- | --- | --- | --- | --- |
| 1 | 2 | 3 | 4 | 5 | 6 |

1. Sé quién soy como persona y qué cosas de la vida son importantes para mí.

| Totalmente en Desacuerdo | En Desacuerdo | Un poco en Desacuerdo | Un poco de Acuerdo | De Acuerdo | Totalmente de Acuerdo |
| --- | --- | --- | --- | --- | --- |
| 1 | 2 | 3 | 4 | 5 | 6 |

1. Las cosas que hago en mi vida tienen sentido y valor.

| Totalmente en Desacuerdo | En Desacuerdo | Un poco en Desacuerdo | Un poco de Acuerdo | De Acuerdo | Totalmente de Acuerdo |
| --- | --- | --- | --- | --- | --- |
| 1 | 2 | 3 | 4 | 5 | 6 |

1. Soy completamente responsable de mi propia vida y bienestar.

| Totalmente en Desacuerdo | En Desacuerdo | Un poco en Desacuerdo | Un poco de Acuerdo | De Acuerdo | Totalmente de Acuerdo |
| --- | --- | --- | --- | --- | --- |
| 1 | 2 | 3 | 4 | 5 | 6 |

**Escala de Evaluación de Recuperación de Maryland – Corta (MARS-12)**

Este cuestionario incluye una serie de frases sobre tus actitudes y creencias acerca de tu salud y bienestar. No hay respuestas correctas o incorrectas.

Lee cada frase y decide tu nivel de acuerdo utilizando la siguiente escala: *1. Nada de acuerdo; 2. Un poco de acuerdo; 3. Algo de acuerdo; 4. Bastante de acuerdo; 5. Muy de acuerdo.*

Por favor, marca sólo un número para cada afirmación y no dejes ninguna sin responder.

| 1  Nada de acuerdo | | 2  Un poco de acuerdo | 3  Algo de acuerdo | 4  Bastante de acuerdo | | | 5  Muy de acuerdo | | | |
| --- | --- | --- | --- | --- | --- | --- | --- | --- | --- | --- |
| 1. | Veo un futuro esperanzador | | | | 1 | 2 | | 3 | 4 | 5 |
| 2. | Creo que las decisiones que tomo en mi vida son buenas | | | | 1 | 2 | | 3 | 4 | 5 |
| 3. | Soy capaz de marcar mis propios objetivos en la vida | | | | 1 | 2 | | 3 | 4 | 5 |
| 4. | Cuando tengo una recaída, estoy seguro/a de que puedo retomar el camino de la recuperación | | | | 1 | 2 | | 3 | 4 | 5 |
| 5. | Estoy convencido/a de que puedo lograr cambios positivos en mi vida | | | | 1 | 2 | | 3 | 4 | 5 |
| 6. | Me siento aceptado/a tal y como soy | | | | 1 | 2 | | 3 | 4 | 5 |
| 7. | Creo que soy una persona fuerte | | | | 1 | 2 | | 3 | 4 | 5 |
| 8. | Me siento bien conmigo mismo/a incluso cuando otros/as me infravaloran por mi estado de salud mental | | | | 1 | 2 | | 3 | 4 | 5 |
| 9. | Puedo tener una vida plena y satisfactoria | | | | 1 | 2 | | 3 | 4 | 5 |
| 10. | Soy optimista y creo que podré resolver los problemas que se me presenten en el futuro | | | | 1 | 2 | | 3 | 4 | 5 |
| 11. | Sé que puedo cambiar aspectos de mi vida a pesar de mi estado de salud mental | | | | 1 | 2 | | 3 | 4 | 5 |
| 12 | Soy responsable de los cambios que hago en mi vida | | | | 1 | 2 | | 3 | 4 | 5 |

**Lista de Empoderamiento de los Países Bajos (NEL)**

A continuación, se muestran 40 afirmaciones. ¿Cuál es tu opinión sobre estas afirmaciones? **Marca la respuesta que mejor coincida con tu opinión en este momento**. La primera respuesta suele ser la mejor. Por lo tanto, no pienses demasiado en una respuesta. Responde todas las preguntas con sinceridad. No des más de una respuesta por cada afirmación.

| 1  Completamente en desacuerdo | | 2  En desacuerdo | 3  Ni de acuerdo ni en desacuerdo | 4  De acuerdo | | | 5  Completamente de acuerdo | | | |
| --- | --- | --- | --- | --- | --- | --- | --- | --- | --- | --- |
| 1. | Los profesionales que me atienden y yo tenemos una buena relación basada en la colaboración. | | | | 1 | 2 | | 3 | 4 | 5 |
| 2. | La sociedad actual tiene en cuenta a las personas con problemas de salud mental. | | | | 1 | 2 | | 3 | 4 | 5 |
| 3. | Creo que puedo significar algo para otras personas. | | | | 1 | 2 | | 3 | 4 | 5 |
| 4. | Tengo un propósito en mi vida. | | | | 1 | 2 | | 3 | 4 | 5 |
| 5. | Las personas de mi entorno me aceptan. | | | | 1 | 2 | | 3 | 4 | 5 |
| 6. | Los/las profesionales que me atienden se basan en mis capacidades en lugar de en mis limitaciones. | | | | 1 | 2 | | 3 | 4 | 5 |
| 7. | Soy capaz de establecer mis límites. | | | | 1 | 2 | | 3 | 4 | 5 |
| 8. | Las personas de mi entorno me escuchan. | | | | 1 | 2 | | 3 | 4 | 5 |
| 9. | Las personas de mi entorno me aceptan como soy. | | | | 1 | 2 | | 3 | 4 | 5 |
| 10. | Sé lo que debo hacer y lo que no debo hacer. | | | | 1 | 2 | | 3 | 4 | 5 |
| 11. | En nuestra sociedad las personas con problemas de salud mental son consideradas ciudadanas de pleno derecho. | | | | 1 | 2 | | 3 | 4 | 5 |
| 12. | Me produce satisfacción que las cosas vayan bien. | | | | 1 | 2 | | 3 | 4 | 5 |
| 13. | Sé qué hacer con los problemas que se me presentan. | | | | 1 | 2 | | 3 | 4 | 5 |
| 14. | Puedo contar con los/las profesionales que me atienden cuando los necesito. | | | | 1 | 2 | | 3 | 4 | 5 |
| 15. | Yo decido cómo llevar las riendas de mi vida. | | | | 1 | 2 | | 3 | 4 | 5 |
| 16. | Cuento con el apoyo de las personas a las que quiero. | | | | 1 | 2 | | 3 | 4 | 5 |
| 17. | Puedo obtener el apoyo adecuado cuando lo necesito. | | | | 1 | 2 | | 3 | 4 | 5 |
| 18. | Estoy decidido/a a seguir adelante. | | | | 1 | 2 | | 3 | 4 | 5 |
| 19. | La sociedad respeta mis derechos como ciudadano/a. | | | | 1 | 2 | | 3 | 4 | 5 |
| 20. | Tengo una vida estructurada. | | | | 1 | 2 | | 3 | 4 | 5 |
| 21. | El rol de persona enferma ya no es central en mi vida. | | | | 1 | 2 | | 3 | 4 | 5 |
| 22. | Me atrevo a pedir ayuda. | | | | 1 | 2 | | 3 | 4 | 5 |
| 23. | La sociedad actual ofrece seguridad y protección a las personas con problemas de salud mental. | | | | 1 | 2 | | 3 | 4 | 5 |
| 24. | Suelo salir de casa y quedar con otras personas con regularidad. | | | | 1 | 2 | | 3 | 4 | 5 |
| 25. | Puedo compartir mis experiencias con otras personas que han tenido experiencias similares. | | | | 1 | 2 | | 3 | 4 | 5 |
| 26. | Sé en qué soy bueno/a. | | | | 1 | 2 | | 3 | 4 | 5 |
| 27. | Tengo una buena relación con las personas de mi entorno. | | | | 1 | 2 | | 3 | 4 | 5 |
| 28. | La sociedad ofrece oportunidades para participar a mi manera. | | | | 1 | 2 | | 3 | 4 | 5 |
| 29. | Los cuidados que recibo son los que necesito en este momento de mi vida. | | | | 1 | 2 | | 3 | 4 | 5 |
| 30. | Siento que formo parte de algo. | | | | 1 | 2 | | 3 | 4 | 5 |
| 31. | Me valoro a mí mismo/a. | | | | 1 | 2 | | 3 | 4 | 5 |
| 32. | Transformo los pensamientos negativos en pensamientos positivos. | | | | 1 | 2 | | 3 | 4 | 5 |
| 33. | Veo cómo la vida me ha convertido en la persona que soy actualmente. | | | | 1 | 2 | | 3 | 4 | 5 |
| 34. | Encuentro paz y seguridad en mi hogar. | | | | 1 | 2 | | 3 | 4 | 5 |
| 35. | Tengo suficientes cosas que hacer cada día. | | | | 1 | 2 | | 3 | 4 | 5 |
| 36. | La sociedad actual no discrimina a las personas con problemas de salud mental. | | | | 1 | 2 | | 3 | 4 | 5 |
| 37. | Hago cosas que son importantes para mí. | | | | 1 | 2 | | 3 | 4 | 5 |
| 38. | Puedo lidiar con mis vulnerabilidades. | | | | 1 | 2 | | 3 | 4 | 5 |
| 39. | Puedo recurrir en las personas de mi entorno. | | | | 1 | 2 | | 3 | 4 | 5 |
| 40. | Me atrevo a confiar en mí mismo/a. | | | | 1 | 2 | | 3 | 4 | 5 |

**Escala de Esperanza Disposicional (DHS)**

Lee cada una de las siguientes frases cuidadosamente. Indica tu acuerdo con cada una de ellas empleando esta escala:

| Totalmente falso | Mayormente falso | Mayormente verdadero | Totalmente verdadero |
| --- | --- | --- | --- |
| 1 | 2 | 3 | 4 |

| 1. | Puedo pensar en muchas salidas cuando me atasco | 1 | 2 | 3 | 4 |
| --- | --- | --- | --- | --- | --- |
| 2. | Persigo mis objetivos enérgicamente | 1 | 2 | 3 | 4 |
| 3. | Me siento cansado la mayor parte del tiempo | 1 | 2 | 3 | 4 |
| 4. | Hay muchas maneras de solucionar un problema | 1 | 2 | 3 | 4 |
| 5. | Me superan fácilmente en una discusión | 1 | 2 | 3 | 4 |
| 6. | Puedo pensar en muchas formas de conseguir las cosas que en la vida son importantes para mí | 1 | 2 | 3 | 4 |
| 7. | Me preocupo por mi salud | 1 | 2 | 3 | 4 |
| 8. | Incluso cuando los demás se desaniman, yo se´ que puedo encontrar una forma de solucionar el problema | 1 | 2 | 3 | 4 |
| 9. | Mis experiencias pasadas me han preparado bien para mi futuro | 1 | 2 | 3 | 4 |
| 10. | He tenido bastante éxito en la vida | 1 | 2 | 3 | 4 |
| 11. | Muchas veces me encuentro preocupado por algo | 1 | 2 | 3 | 4 |
| 12. | Consigo los objetivos que me propongo | 1 | 2 | 3 | 4 |

**Escala Multifactorial de Apoyo Social Percibido (MSPSS)**

Lee cada una de las siguientes frases cuidadosamente. Indica tu acuerdo con cada una de ellas empleando esta escala:

| 1 | 2 | 3 | 4 | 5 | 6 | 7 |
| --- | --- | --- | --- | --- | --- | --- |
| Totalmente en desacuerdo | Bastante en desacuerdo | Más bien en desacuerdo | Ni de acuerdo ni en desacuerdo | Más bien de acuerdo | Bastante de acuerdo | Totalmente de acuerdo |

| 1. | Hay una persona que está cerca cuando estoy en una situación difícil | 1 | 2 | 3 | 4 | 5 | 6 | 7 |
| --- | --- | --- | --- | --- | --- | --- | --- | --- |
| 2. | Existe una persona especial con la cual yo puedo compartir penas y alegrías | 1 | 2 | 3 | 4 | 5 | 6 | 7 |
| 3. | Mi familia realmente intenta ayudarme | 1 | 2 | 3 | 4 | 5 | 6 | 7 |
| 4. | Obtengo de mi familia la ayuda y el apoyo emocional que necesito | 1 | 2 | 3 | 4 | 5 | 6 | 7 |
| 5. | Existe una persona que realmente es una fuente de bienestar para mí | 1 | 2 | 3 | 4 | 5 | 6 | 7 |
| 6. | Mis amigos realmente tratan de ayudarme | 1 | 2 | 3 | 4 | 5 | 6 | 7 |
| 7. | Puedo contar con mis amigos cuando las cosas van mal | 1 | 2 | 3 | 4 | 5 | 6 | 7 |
| 8. | Yo puedo hablar de mis problemas con mi familia | 1 | 2 | 3 | 4 | 5 | 6 | 7 |
| 9. | Tengo amigos con los que puedo compartir las penas y alegrías | 1 | 2 | 3 | 4 | 5 | 6 | 7 |
| 10. | Existe una persona especial en mi vida que se preocupa por mis sentimientos | 1 | 2 | 3 | 4 | 5 | 6 | 7 |
| 11. | Mi familia se muestra dispuesta a ayudarme para tomar decisiones | 1 | 2 | 3 | 4 | 5 | 6 | 7 |
| 12. | Puedo hablar de mis problemas con mis amigos | 1 | 2 | 3 | 4 | 5 | 6 | 7 |
